# Risk factors for metachronous adenoma in the residual colon of patients undergoing curative surgery for colorectal cancer

**DOI:** 10.1007/s00384-017-2881-x

**Published:** 2017-08-21

**Authors:** Abhilasha Patel, Nigel Williams, Nicholas Parsons, Omar Ali, Francesca Peters, Reesha Ranat, Jasmine Shah, Emma Spector, Ramesh P. Arasaradnam

**Affiliations:** 1 Department of Colorectal Surgery, University Hospitals of Coventry and Warwickshire NHS Trust, University of Coventry, Clifford Bridge Road, Coventry, CV2 2DX UK; 2 Statistics and Epidemiology, University of Warwick, Coventry, CV4 7AL UK; 3 University of Warwick, Coventry, CV4 7AL UK; 4 University Hospitals of Coventry and Warwickshire NHS Trust, University of Coventry, Clifford Bridge Road, Coventry, CV2 2DX UK

**Keywords:** Colorectal cancer, Polyps, Adenomas, Synchronous, Metachronous

## Abstract

**Purpose:**

Adenoma detection in colorectal cancer survivors is poorly characterised with insufficient evidence to inform frequency of surveillance schedule. The aim of this study was to examine adenoma incidence and recurrence in patients who have undergone colorectal cancer resection with curative intent. Survival outcomes were compared to determine if the presence of adenomas could be used to identify patients at higher risk of local recurrence.

**Methods:**

This is a retrospective observational cohort study at a single tertiary institution between 2006 and 2012. Five hundred fifteen consecutive patients with stage I–III colorectal cancer who had preoperative colonoscopy and curative surgery were included (median follow-up 56 months (36–75 months).

**Results:**

In total, 352/515 (68%) patients underwent postoperative surveillance colonoscopy in the first 5 years after resection. Male gender was associated with greater risk of detecting synchronous adenoma at index colonoscopy or in the resection specimen (OR 2.35, *p* < 0.001). In the first 5 years after cancer surgery, synchronous adenoma, male gender and right sided primary tumour were independent predictors of metachronous lesions (OR 2.13, *p* = 0.009; OR 2.07, *p* = 0.027 and OR 2.34, *p* = 0.004, respectively). Presence of synchronous or metachronous adenoma had no impact upon incidence of local recurrence, overall or disease free survival.

**Conclusions:**

Patients with synchronous adenoma remain at high risk of adenoma recurrence despite undergoing colonic resection and should be considered for early endoscopic surveillance. Men and those undergoing right-sided resection have a higher risk of metachronous adenoma in the long term and may benefit from more frequent endoscopic surveillance post resection.

## Introduction

Patients diagnosed with colorectal cancer (CRC) remain at higher risk of developing recurrent disease or metachronous cancer compared to the general population [1]. Early detection of recurrence, prior to it becoming symptomatic, is associated with improved chances of cure and survival [2]. At present, there is considerable variation in recommended surveillance schedules for these patients. In the UK, the National Institute for Clinical Excellence recommends colonoscopy at 1 year after resection [3]. If this demonstrates adenomas, frequency of subsequent endoscopic examination is determined using conventional adenoma parameters such as size, number and grade of dysplasia [4]. The US Multi-Society Task Force [5] and ASCO [6] both recommend colonoscopy 1 year after resection. However, they differ in subsequent surveillance as the former suggests further colonoscopy in 3 years whereas the latter proposes a 5-year interval. Both indicate that if any post-resection colonoscopy is positive, subsequent endoscopic examination should be based on post-polypectomy surveillance guidelines [4]. These guidelines describe surveillance in patients who do not have cancer and require inspection of their entire colon. In comparison, patients who have undergone surgery may have their risk of adenoma reduced as they are left with a shorter segment of bowel. This would imply that it is inappropriate to utilise conventional adenoma parameters to risk stratify these patients, and other clinical factors should be sought to identify those that are at higher risk of developing adenomas in their residual colon.

The rate of metachronous adenoma (MA) in CRC survivors varies between 8 and 46% [7–11] depending upon the definition of MA, the surveillance frequency and loss to follow-up. There have been several reports of a link between synchronous adenoma (SA) and risk of both MA and subsequent cancer [9, 12–14], implying that SAs may confer additional risk of neoplasia.

The primary aim of this study was to examine adenoma incidence and recurrence in the residual colon after surgery to determine if there were any clinical or pathological risk factors that could be utilised to inform surveillance schedule. Secondly, the relationship between adenomas and survival was explored to ascertain if the development of adenomas indicates a higher risk of neoplastic transformation in the residual colon as measured by loco-regional recurrence rather than de novo cancer formation. This is based upon the field cancerisation theory [15], which proposes that cancer develops within fields of genetically altered, though macroscopically normal mucosa. Adenoma occurrence in the residual colon after surgery could signify genetic alterations in the underlying macroscopically normal mucosa which could contribute to loco-regional recurrence. Thus, the findings of this study could be utilised to counsel patients with colorectal cancer who are found to have adenomas on surveillance colonoscopy regarding their risk of developing local recurrence and its effect upon survival.

## Methods

Institutional Board Approval was obtained from the research and development department at our hospital. Patients with stage I–III CRC undergoing surgical resection with curative intent between 2006 and 2012 were included. Only those that had undergone preoperative colonoscopy were included. The following were excluded: stage IV disease at presentation, cancer confined to adenoma, genetic predisposition to CRC such as familial adenomatous polyposis (FAP) or hereditary non-polyposis colorectal cancer (HNPCC), underlying inflammatory bowel disease, synchronous tumour and patients who had total colectomy.

Data on patient demographics, operative details, pathological findings, endoscopic observations and survival was obtained from hospital electronic records.

The departmental policy for CRC follow-up involves clinic review with routine blood tests including biannual serum carcinoembryogenic antigen (CEA) in the first 2 years followed by annual tests for the next 3 years. All patients undergo inspection of their anastomosis within 1 year of surgery with either a flexible sigmoidoscopy or colonoscopy depending upon site of surgery. A further colonoscopy is performed at 5 years unless there are positive findings in the first postoperative investigation, in which case, interval colonoscopy is performed as per the UK polyp surveillance guidelines [4]. A CT scan is performed at three and 5 years after surgery.

### Definitions of clinical groups

The SAs group consisted of patients with adenomas detected at index colonoscopy or patients in whom adenomas were discovered in the resection specimen by the pathologist. A histological diagnosis was necessary to be allocated to the SA group. All adenomas detected in the preoperative setting were removed endoscopically. Any adenomas identified after surgery on surveillance colonoscopy were classified as MAs. Outcomes in the first 5 years after surgery were calculated based on cumulative colonoscopy findings over this period of time.

Overall survival (OS) was calculated from the date of surgery (DOS) to the date when the patient was last seen alive in the hospital. Disease free survival (DFS) was estimated between the DOS and the date when the patient was last seen without disease recurrence in the oncology or surgical clinic. The date when local recurrence or metastasis was first detected on imaging/endoscopy was utilised to estimate DFS.

Local recurrence was defined as either soft tissue growth at the anatomical site of previous resection on radiological imaging or intraluminal recurrence at the anastomotic site on colonoscopy.

### Statistical data analysis

Differences in categorical clinical variables amongst the different groups (SA versus non-SA, MA versus non MA) were tested with the chi-squared test. A *p* value of < 0.05 was considered to be statistically significant. A multiple logistic regression model was built to determine which factors affect incidence of metachronous adenomas in CRC survivors. The following variables were assessed: age (<60 years versus > 60 years), sex, tumour location (right versus left colon where the former includes all tumours proximal to the splenic flexure), T stage, N stage, mucinous content (any tumour containing mucinous content versus those without) and presence of SA. It was decided, a priori, to include both age and sex in the minimum model, as these were considered highly likely to be important predictors of outcome or moderators for the effects of the other variables under test. The other variables that were included in the model comprised those with a *p* value of < 0.05 after the initial screening in univariate analyses. The Kaplan-Meier method was used to derive overall and disease free survival. Difference in survival between groups was tested using the log-rank test.

## Results

In total, 701 patients with stage I–III colorectal cancer undergoing surgical resection with curative intent were eligible for inclusion, of which, 515 (73%) underwent preoperative colonoscopy. The clinical and pathological details are given in Table 1. Men with early tumours who underwent surgery in an elective setting were more likely to have undergone preoperative colonoscopy. Overall and disease free survival were lower in those who had no preoperative endoscopic examination.

**Table 1:** Clinicopathological details of patients who had preoperative colonoscopy compared to those that did not undergo endoscopic examination (*n* = 701)

	Preoperative colonoscopy patients (*n* = 515)	No colonoscopy patients (*n* = 186)	*p* value
Age			
< 60 years	106/515	32/186	0.335
M:F	299:216	83:103	0.002**
Right-sided tumour	210/515	91/186	0.058
Mode of surgery			
Elective	304	76	< 0.001
Emergency	8	32	
Scheduled	163	34	
Urgent	40	41	
Operative procedure			
Anterior resection	180	29	
Abdominoperineal resection	41	6	
Hartmann’s procedure	27	31	
Left hemicolectomy	31	12	
Sigmoid colectomy	19	8	
Transverse colectomy	2	1	
Extended right hemicolectomy	18	6	
Right hemicolectomy	195	90	
Laparotomy	2	3	
T stage			
pT0-pT2	106	12	< 0.001
pT3-pT4	409	174	
N stage			
pN0	346	101	0.002
pN1-pN2	169	85	
Adenoma post resection			
0–24 months FU	50/287	15/69	0.391
0–60 months FU	66/352	24/79	0.031*
Survival			
3-year OS	91%	74%	< 0.001**
3-year DFS	86%	62%	< 0.001**

Asterisk denotes statistically significant results

### Rate of missed adenoma at preoperative colonoscopy

There were 60 (12% of total) patients who had adenomas in the resection specimen which were not identified endoscopically.

Patients in whom adenomas were missed on colonoscopy were more likely to have right-sided tumours compared to those in whom adenomas were detected at colonoscopy (63 versus 35%, *p* < 0.001, chi-squared test). There was no association with other clinical factors tested.

### Postoperative endoscopic surveillance

**Fig. 1:**
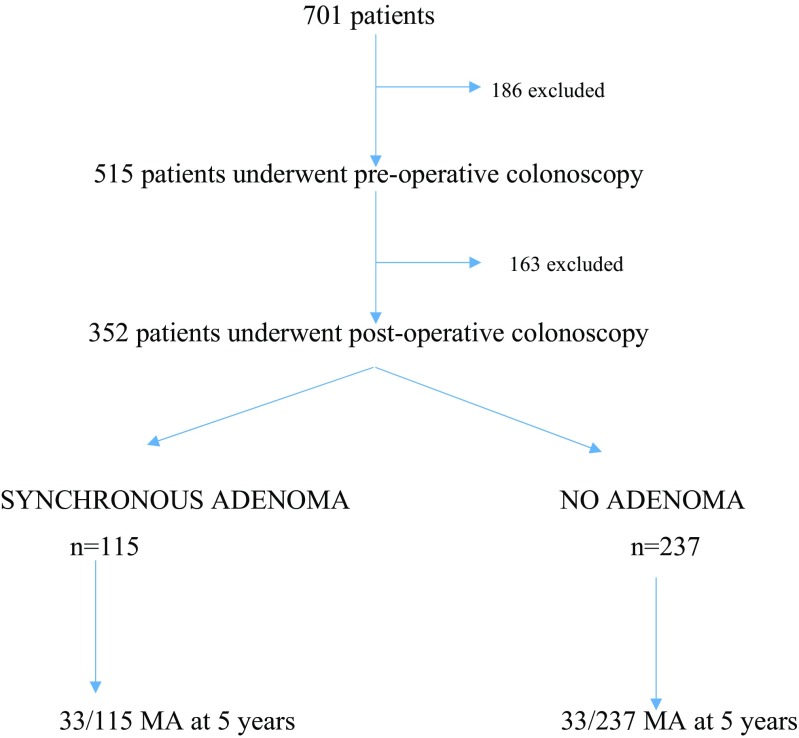
Flow diagram showing patients included in the analysis

Within the first 5 years after surgery, 352/515 (68%) patients underwent colonoscopy and have been included in subsequent analysis (see Fig. 1). An additional 43 patients had flexible sigmoidoscopy. The remaining patients did not undergo any endoscopic examination during the postoperative period. Women and older patients, with more advanced tumours, were less likely to undergo endoscopic surveillance (see Table 2), and these patients had a significantly lower overall and disease free survival.

**Table 2:** Clinical, operative and pathological details of patients undergoing endoscopic surveillance post cancer resection compared to patients who did not undergo postoperative colonoscopy (*n* = 515)

	Postoperative colonoscopy patients (*n* = 352)	No surveillance patients (*n* = 163)	*p* value
Age			
< 60 years	93/352	13/163	< 0.001**
M:F	217:135	82:81	0.017*
Right-sided tumour	137/352	73/163	0.212
Mode of surgery			
Elective	205	99	0.358
Emergency	4	4	
Scheduled	118	45	
Urgent	25	15	
T stage			
pT0-pT2	75	31	0.013
pT3-pT4	277	132	
N stage			
pN0	248	98	0.027
pN1-pN2	104	65	
Survival			
3-year OS	96%	78%	< 0.001
3-year DFS	90%	74%	< 0.001

Asterisk denotes statistically significant results

### Factors associated with synchronous adenomas

Of the 352 patients who underwent postoperative colonoscopy, 115 (33%) had synchronous adenomas. Male gender was significantly associated with the presence of SAs (88/115 (77%) versus 129/237 (54%), *p* < 0.001). There were no significant differences in other clinical and pathological parameters recorded (see Table 3).

**Table 3:** Clinical, operative and pathological details of SA and non-SA patients (*n* = 352)

	SA patients (*n* = 115)	Non-SA patients (*n* = 237)	*p* value
Age			
< 60 years	23	70	< 0.071
> 60 years	92	167	
M:F	88:27	129:108	< 0.001***
Right-sided tumour	49/115	88/237	0.352
T stage			
pT0-pT2	26	49	0.679
pT3-pT4	89	188	
N stage			
pN0	165	83	0.709
pN1-pN2	72	32	
Metachronous adenomas			
0–24 months FU	28/100	22/187	< 0.001***
0–60 months FU	33/115	33/237	< 0.001***
Survival			
5-year overall survival	88%	93%	0.162
5-year disease free survival	83%	83%	0.695

Asterisk denotes statistically significant results

### Factors associated with metachronous adenoma

In the first 5 years after surgery, 66/352 (19%) patients who underwent colonic examination had developed MA. The presence of SA was associated with a higher incidence of MA (29 versus 14%, *p* = 0.001). In a multivariate logistic regression model, the independent predictors of MA by 60 months were SA at diagnosis, male gender and a right-sided tumour (see Table 4). Patients in whom SAs were detected in the resection specimen had a similar 5-year risk of subsequent MA compared to those in whom adenomas were detected preoperatively (11/36 (31%) versus 22/79 (28%), *p* = 0.825).

**Table 4:** Univariate and multivariate factors that predict the development of MAs in CRC patients 0–60 months after surgery (*n* = 352)

Variable	MA present	Univariate *p* value	Odds ratio	95% CI	Multivariate *p* value
Age	< 60 yrs-17/93	1.000	1.182	0.621–2.253	0.610
	> 60 yrs-49/259				
Sex	49/217 M	0.024	2.064	1.088–3.916	0.027*
	17/135 F				
Site of tumour	35/137 R	0.012	2.341	1.316–4.162	0.004**
	31/215 L				
Synchronous adenoma	33/237 none	0.001	2.133	1.205–3.775	0.009*
	33/115 present				

Asterisk denotes statistically significant results

The presence of advanced synchronous adenoma (greater than 10 mm in size, presence of high-grade dysplasia or more than 3 adenomas) did not predict higher incidence of MA at 5 years (14/57 (25%) with advanced SA versus 52/295 (18%) with simple SA, *p* = 0.265, chi-squared test).

If patients who are found to have MA within 12 months of surgery are excluded as these patients may actually have SA that were missed at index colonoscopy, SA at diagnosis still predicts a higher risk of developing MA between 12 and 60 months after the resection (33 versus 15%, *p* = 0.001).

### Adenoma characteristics

The characteristics of metachronous adenomas detected during the study period are given in Table 5. There were 14/66 (21%) patients with advanced adenomas (greater than 10 mm or high-grade dysplasia).

**Table 5:** Metachronous adenoma characteristics

	Right-sided resection	Left-sided resection
Site		
Caecum	0	9
Ascending	0	4
Hepatic	0	6
Transverse	4	11
Splenic	2	6
Descending	6	4
Sigmoid	10	2
Rectosigmoid	2	0
Rectum	13	5
Anastomosis	8	2
Mean size (mm)	7	6
Type of adenoma		
Tubular adenoma	34	31
Serrated adenoma	2	2
Tubulovillous adenoma	6	5
Grade of dysplasia		
Low	41	37
High	1	1
Adenocarcinoma	0	0

### Adenomas and local recurrence

Patients with SA had lower local recurrence than non-SA patients (1/115 (0.8%) versus 11/237 (4.6%), *p* = 0.004, chi-squared test). However, there was no difference in local recurrence between those who developed MAs compared to those that did not (1/66 (1.5%) versus 11/286 (3.8%), *p* = 0.705, chi-squared test).

### Adenomas and overall and disease free survival

There was no difference in OS or DFS between SA and non-SA patients (see Table 3). Patients who developed MAs had similar outcomes to patients who did not (5-year overall survival 90% versus 92%, *p* = 0.544).

## Discussion

Endoscopic surveillance strategies vary considerably post cancer resection. Current guidelines are based upon risk stratification using adenoma characteristics as described in the literature on adenoma recurrence in non cancer patients. The key difference is that those who have undergone surgery have a shorter segment of residual colon, and it is unclear how likely this segment of bowel is to develop MAs. Our study has highlighted that in the first 5 years after surgery, presence of SA, male gender and right-sided resection are independent predictors of subsequent MA suggesting that these patient factors are important in identifying those at increased risk. Patients with ‘missed’ adenomas detected in the resection specimen but not identified at time of colonoscopy have a similar increased risk of metachronous adenoma as patients with adenomas that were noted endoscopically. Furthermore, there is no relationship between development of MAs and loco-regional recurrence highlighting that adenoma recurrence is a poor surrogate marker for identifying those at risk of recurrent disease. These patients will remain at risk of de novo cancer formation and given that this study only examined outcomes in the first 5 years after surgery; it is not possible to evaluate how MAs impact on development of metachronous cancer.

The incidence of SA (35%) and MA (19%) in this study is comparable to previous reports which quote rates varying between 8 and 46% [7–11]. The large range described in the literature reflects the lack of agreed consensus on how frequently endoscopic surveillance should be performed in these patients. Chemoprevention trials where adenomas are utilised as a surrogate marker of neoplastic risk illustrate higher incidence of both SA and MA [16], particularly with advanced adenoma [17], indicating that more intensive, focussed endoscopic evaluation results in higher detection of these pre-neoplastic lesions.

In our study, SAs were more likely to be detected in men; there were no other clinical variables that were found to differ between the two groups. This compares to the existing literature where other factors such as obesity [18], mucinous cancer [19], proximal tumours [20, 21] and stage II disease [19] have all been associated with the presence of synchronous lesions.

In terms of factors that discriminate CRC patients that develop metachronous lesions, the presence of SA at diagnosis, male gender and right-sided primary tumour were independent predictors of MAs. Patients with missed adenomas on preoperative investigation were also more likely to have MAs. This suggests that it is important to identify and record adenomas present in the resection specimen as they are indicative of a higher risk of MA in CRC survivors. In a large population based study from Netherlands, 43% metachronous cancers were attributed to ‘missed’ lesions and only 5.4% resulted from de novo cancer formation [22]. Over a longer period of time, MAs that are detected are proportionately less likely to represent missed adenomas and more likely to reflect de novo neoplasia. This implies that SAs confer a higher risk of MA. A recent Japanese study on 309 patients reported a significantly shorter adenoma free 5-year survival in patients with SA compared to those without [23]. Based on the findings, the authors concluded that the age and presence of SA were independent predictors of MA and described a nomogram using these variables that was capable of predicting development of MA. In our study, adenoma incidence was recorded as a binary outcome and not calculated using Kaplan-Meier analysis which may explain the different conclusion regarding age as a discriminant factor.

In the present study, there were distinct differences in the relationship between SA and MA with tumour location. SAs in patients with right-sided tumours were associated with a higher incidence of MA, in comparison to left-sided tumours, where presence of SA did not predict a higher risk of MA. This is supported by previous studies which have also demonstrated that both MA and metachronous cancer occur more frequently in patients with a proximal tumour [24, 25]. A more recent study, however, has challenged this view by describing a higher rate of MAs in patients with distal tumours and postulated that removal of the right colon protects against development of MAs which usually occur proximal to the primary cancer [26]. The observed association between proximal tumours and adenomas was previously believed to arise from cases with undiagnosed HNPCC and microsatellite instability (MSI) who are more likely to present with multiple lesions [27, 28]. Yet, there have recently been reports to suggest that the risk of MAs is not linked to microsatellite instability [12] nor is the incidence of MA in patients with various degrees of MSI any different [29]. Thus, the higher incidence of MAs with proximal tumours noted in our study may reflect other differences in tumour biology aside from microsatellite instability, such as methylator phenotype.

Importantly, no adenoma characteristics of those synchronous lesions detected at time of diagnosis were predictive of MAs, both over short and long-term follow-up. This contradicts previous reports where only advanced adenomas (more than 1 cm, villous architecture or high-grade dysplasia) were associated with increased risk of finding MAs [30]. A non-advanced adenoma has been shown to have very low risk of forming an advanced adenoma at follow-up [9]. Our findings suggest that risk stratification based on adenoma characteristics at index colonoscopy or subsequently may not be appropriate in CRC survivors.

Importantly, our study did not demonstrate any differences in local recurrence, DFS and OS in patients with SAs compared to those without. There are several possible explanations which may account for these findings. A recent randomised controlled trial from China revealed that there was no difference in survival between patients undergoing intensive endoscopic post-resection surveillance compared to standard surveillance [31]. Several other trials and meta-analyses have shown improved overall survival but failed to demonstrate any differences in disease free survival with intensive post-resection surveillance strategies questioning the importance of adenoma detection and removal in these patients [32–34]. Furthermore, those studies where improved overall survival has been observed have been criticised for choosing healthier patients for endoscopic examination and using a less intensive follow-up strategy for patients who are deemed medically unfit for intervention should recurrent disease be detected. There may be little survival benefit in removing metachronous lesions in the elderly population who undergo cancer resection, and these patients are more likely to develop disease recurrence rather than de novo cancer [9]. Others have proposed that improved survival seen in patients who undergo intensive surveillance is the result of ‘lead time bias’ and higher rates of intervention for disease recurrence as was demonstrated in the recently published FACS trial [2]. This indicates that recurrent adenoma formation poses a lesser threat to survival than disease recurrence or distant spread in colorectal cancer survivors.

There are several limitations to our study that require consideration. Firstly, this is a retrospective study where there was some missing data; however, this contributed to less than 1% of the total dataset and given the number of patients included in the study is unlikely to have had a significant impact on outcome. There were some patients (32%) who did not undergo any postoperative endoscopic examination; however, the analysis investigating factors that are predictive of adenoma recurrence excluded these patients. One could argue that patients who underwent colonoscopy and had polyps were more likely to undergo subsequent colonoscopy increasing the number of MAs detected in this group. However, the primary outcome was binary in nature; therefore, difference in surveillance strategy is unlikely to impact upon the results.

In conclusion, synchronous lesions at time of diagnosis help to identify individuals at increased risk of MA within the first 5 years after surgery. Men with proximal tumours who present with SAs also have a high risk of MA, and a more intensive surveillance strategy may be appropriate in this group. Other patients presenting with SA can safely be counselled that their risk of disease recurrence or survival is not increased by the presence of adenomas at index colonoscopy or subsequent endoscopic surveillance.
